# The first chromosome-level *Fallopia multiflora* genome assembly provides insights into stilbene biosynthesis

**DOI:** 10.1093/hr/uhad047

**Published:** 2023-03-15

**Authors:** Yujiao Zhao, Zhengyang Yang, Zhongren Zhang, Minzhen Yin, Shanshan Chu, Zhenzhen Tong, Yuejian Qin, Liangping Zha, Qingying Fang, Yuan Yuan, Luqi Huang, Huasheng Peng

**Affiliations:** School of Pharmacy, Anhui University of Chinese Medicine, Hefei 230012, China; Anhui Province Key Laboratory of Research & Development of Chinese Medicine, Hefei 230012, China; School of Pharmacy, Anhui University of Chinese Medicine, Hefei 230012, China; Novogene Bioinformatics Institute, Beijing 301700, China; School of Pharmacy, Anhui University of Chinese Medicine, Hefei 230012, China; School of Pharmacy, Anhui University of Chinese Medicine, Hefei 230012, China; Anhui Province Key Laboratory of Research & Development of Chinese Medicine, Hefei 230012, China; School of Pharmacy, Anhui University of Chinese Medicine, Hefei 230012, China; School of Pharmacy, Anhui University of Chinese Medicine, Hefei 230012, China; School of Pharmacy, Anhui University of Chinese Medicine, Hefei 230012, China; Anhui Province Key Laboratory of Research & Development of Chinese Medicine, Hefei 230012, China; School of Pharmacy, Anhui University of Chinese Medicine, Hefei 230012, China; Anhui Province Key Laboratory of Research & Development of Chinese Medicine, Hefei 230012, China; National Resource Center for Chinese Materia Medica, China Academy of Chinese Medical Sciences, Beijing 100700, China; National Resource Center for Chinese Materia Medica, China Academy of Chinese Medical Sciences, Beijing 100700, China; School of Pharmacy, Anhui University of Chinese Medicine, Hefei 230012, China; National Resource Center for Chinese Materia Medica, China Academy of Chinese Medical Sciences, Beijing 100700, China; Research Unit of DAO-DI Herbs, Chinese Academy of Medical Sciences, 2019RU57, Beijing 100700, China

## Abstract

*Fallopia multiflora* (Thunb.) Harald, a vine belonging to the Polygonaceae family, is used in traditional medicine. The stilbenes contained in it have significant pharmacological activities in anti-oxidation and anti-aging. This study describes the assembly of the *F. multiflora* genome and presents its chromosome-level genome sequence containing 1.46 gigabases of data (with a contig N50 of 1.97 megabases), 1.44 gigabases of which was assigned to 11 pseudochromosomes. Comparative genomics confirmed that *F. multiflora* shared a whole-genome duplication event with Tartary buckwheat and then underwent different transposon evolution after separation. Combining genomics, transcriptomics, and metabolomics data to map a network of associated genes and metabolites, we identified two *FmRS* genes responsible for the catalysis of one molecule of *p*-coumaroyl-CoA and three molecules of malonyl-CoA to resveratrol in *F. multiflora*. These findings not only serve as the basis for revealing the stilbene biosynthetic pathway but will also contribute to the development of tools for increasing the production of bioactive stilbenes through molecular breeding in plants or metabolic engineering in microbes. Moreover, the reference genome of *F. multiflora* is a useful addition to the genomes of the Polygonaceae family.

## Introduction

*Fallopia multiflora* (Thunb.) Harald is a perennial vine belonging to the Polygonaceae family. It is primarily found in the southern parts of China’s Qinling Mountains-Huaihe River [1]. *F. multiflora* has high economic value. For almost 1000 years, the root tuber, also described as ‘Heshouwu’ in Chinese, has been incorporated in numerous formulae in traditional Chinese medicine (TCM) [2, 3] and recorded in the Japanese and the Korean *Pharmacopoeia* [4, 5]. Clinically, it is usually used for detoxification, carbuncles elimination, and intestines moistening, as well as a laxative and antimalarial agent [3]. Its processed products (‘Zhi Heshouwu’ in Chinese) tone the liver and kidneys and help in maintaining hair colour [3]. Additionally, it has been widely used in TCM to treat allergies and alopecia in America, Australia, and Europe [6]. Moreover, the sales of *Heshouwu*-related preparations and washing products are considerable; however, because of its hepatotoxicity, *F. multiflora* has attracted extensive attention and research interest.

The chemical components isolated from *F. multiflora* include stilbenes, anthraquinones, flavones, phenols, and other important compounds [2, 7–9]. Among these, stilbenes are the main chemical constituents and one of the key active components, which mainly include 2,3,5,4-tetrahydroxy stilbene-2-O-β-D-glucoside (THSG), resveratrol, and polydatin. Stilbene compounds are considered as plant phytoalexins and are involved in plantprotection against pests, pathogens, and abiotic stresses [2]. Modern medical research indicates that stilbenes from *F. multiflora* exert anti-aging, anti-depression, anti-inflammatory, anti-hyperlipidemic, immunomodulatory, antioxidant, anti-osteoporosis, and hepatoprotective effects [2, 10]. Specifically,resveratrol has been proved to reduce the risk of various diseases, including cardiovascular disease, Alzheimer’s disease, and cancer [11–13]. Due to the wide application of resveratrol in the medicineand food industries, its demand continues to increase. However, resveratrol is naturally synthesized in only a few plants, and its content varies greatly. The biosynthetic pathway of resveratrolin *Vitis vinifera* is currently the most intensively studied stilbene pathway. It is a small branch of the pathway for the synthesis of phenylalanine that is also associated with the flavonoid synthetic pathway. Stilbene synthase (STS) is a type III polyketide synthase (PKS) that contributes significantly to stilbene synthesis [14].The enzyme catalyzes one molecule of 4-coumarinyl coenzyme A and three molecules of malonyl coenzyme A to produceresveratrol [14]. Notably, the research on biosynthetic pathway of resveratrol in *F. multiflora* remains lagging behind, and the lack ofgenomic data seriously hinders the construction of the pathway,as genomic data are crucial for advancing our understanding of stilbene biosynthesis and diversity in plants.

Here, the assembly of the chromosomal-level *F. multiflora* genome is described. This was done using a combination of PacBio, 10X Genomics*,* Illumina, and high-throughput chromosome conformation capture (Hi-C) technologies, followed by genomic comparisons and phylogenetic analyses. The findings suggested that *F. multiflora* and Tartary buckwheat had shared a whole-genome duplication (WGD) during their evolution. Additionally, we characterized *resveratrol synthase* (*RS*) genes that greatly contribute to resveratrol biosynthesis in *F. multiflora*. The *F. multiflora* reference genome offers insight into the evolution of dicotyledons and will be useful for future genetic investigations and medicinal uses of the Polygonaceae.

## Results

### Genome sequencing, assembly, and annotation

**Figure 1:**
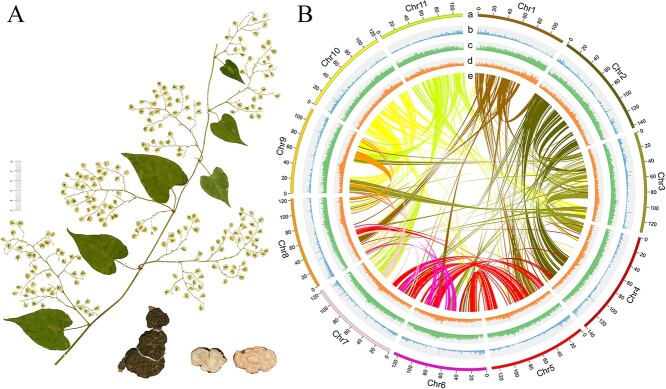
The plant *Fallopia multiflora* and genome assembly. **A** Morphology of the stem and leaf in fruit stage, root tuber, and cross-section of root tuber displayed. **B** Distribution of *F. multiflora* genomic features. (i) The 11 assembled chromosomes, (ii) the gene density, (iii) the TE density, (iv) the GC content, and (v) each line in the circle’s center connects two homologous genes.

As shown by K-mer distribution and flow cytometry, we estimated the *F. multiflora* genome’s size (2n = 2x = 22) to be ~1.46 gigabases (Gb) with a high heterozygosity level of 0.64% and 70.65% repetition, which suggested a complicated genome assembly (Fig. S1 and Table S1, see online supplementary material). We sequenced the *F. multiflora* genome using Illumina (San Diego, CA, USA), PacBio (Menlo Park, CA, USA), and 10X Genomics (San Diego, CA, USA) systems. This results in an assembly of 1.44 Gb containing 1211 contigs and 1.97 megabase (Mb) contig N50 (Table 1; Table S2, see online supplementary material). Further refinement of the *F. multiflora* assembly with Hi-C data resulted in a refined assembly comprising 392 scaffolds with an N50 of (122.50 Mb) for the scaffolds as presented in Table 1. Furthermore, 1.44 Gb (96.12%) of the assembly was distributed across 11 pseudomolecules at the chromosome level (Fig. 1 and Table 1; Fig. S2 and Tables S3 and S4, see online supplementary material).

**Table 1:** An overview of the assembly and annotation of the *Fallopia multiflora* genome

Items	Number	Size (bp)
Genome assembly		
Total contigs	1595	1 439 539 278
Contig N50	222	1 688 979
Contig N90	834	500 000
Total scaffolds	392	1 442 311 844
Scaffold N50	6	122 496 873
Scaffold N90	11	112 054 525
Pseudochromosomes	11	1 386 380 594
Genome annotation		
Repetitive sequences	67.69%	976 298 377
Noncoding RNAs		
Protein-coding genes	35 575	

To evaluate the assembly, short high-quality reads were then mapped to the assembled genome, resulting in the mapping of 99.44% of these reads with an overall coverage of 99.80% (Table S5, see online supplementary material). Additionally, analysis of the assembly using CEGMA showed that it covered 96.37% of the conserved eukaryotic core genes, while BUSCO (Benchmarking Universal Single-Copy Orthologs) indicated that the genome was 96.5% complete (Table S6, see online supplementary material). These results confirmed the high-quality assembly of the *F. multiflora* genome at the chromosome level.

It was predicted that the genome contained 35 926 protein-coding genes. These genes were then annotated using RNA sequencing, de novo and homology analyses (Table S7, see online supplementary material). The average predicted lengths of genes were 3179 bp, with average exon and intron lengths of 226 and 542 bp, respectively (Table S7, see online supplementary material). Among the protein-coding genes, 35 575 (99.00%) showed homology to characterised genes based on BLAST searches, and 24 570 (68.4%) and 32 387 (90.1%) were assigned to Gene Ontology (GO) terms and Kyoto Encyclopedia of Genes and Genomes (KEGG) pathways, accordingly (Table S8, see online supplementary material). These findings demonstrated the great reliability of predicted genes in the *F. multiflora* genome. Moreover, we annotated noncoding RNAs, including microRNA (1202), transfer RNAs (3500), ribosomal RNAs (1298), and small nuclear RNAs (2851) as illustrated in Table S9 (see online supplementary material).

### Gene family evolution and phylogenetic analysis

The evolutionary relations among *F. multiflora*, *Fagopyrum tataricum*, *Beta vulgaris* from Caryophyllales, three rosid species (*Arabidopsis thaliana*, *Arachis duranensis*, and *V. vinifera*), four asterid species (*Solanum lycopersicum*, *Daucus carota*, *Lactuca sativa*, and *Salvia miltiorrhiza*), and a monocot species (*Oryza sativa*) (Table S10, see online supplementary material) were investigated. OrthoMCL was used for orthologus clustering analysis and gene family clustering analysis. Briefly, 27 474 *F. multiflora* genes (76.47%) clustered into 26 741 gene families (Fig. S3, see online supplementary material), which included 6034 families of genes that were distributed through all 11 species and families (1031) that were particular to *F. multiflora.* We then compared the gene families among the three Caryophyllales species, finding that 9583 gene families were shared in *F. multiflora*, *F. tataricum*, and *B. vulgaris* (Fig. S4, see online supplementary material). Compared with two other Caryophyllales species [*F. tataricum* (980) and *B. vulgaris* (996)], *F. multiflora* had a higher number of unique gene families (1436) (Fig. S4, see online supplementary material). Among the pairwise combinations of the three species, *F. multiflora* and *F. tataricum* (12696) shared the highest gene family clusters. The GO and KEGG enrichment results indicated that the gene families specific to *F. multiflora* were involved in a variety of biological activities, including proteolysis, peptidase activity, photosynthesis, protein processing in the endoplasmic reticulum, and RNA transport (Fig. S5 and S6, see online supplementary material).

**Figure 2:**
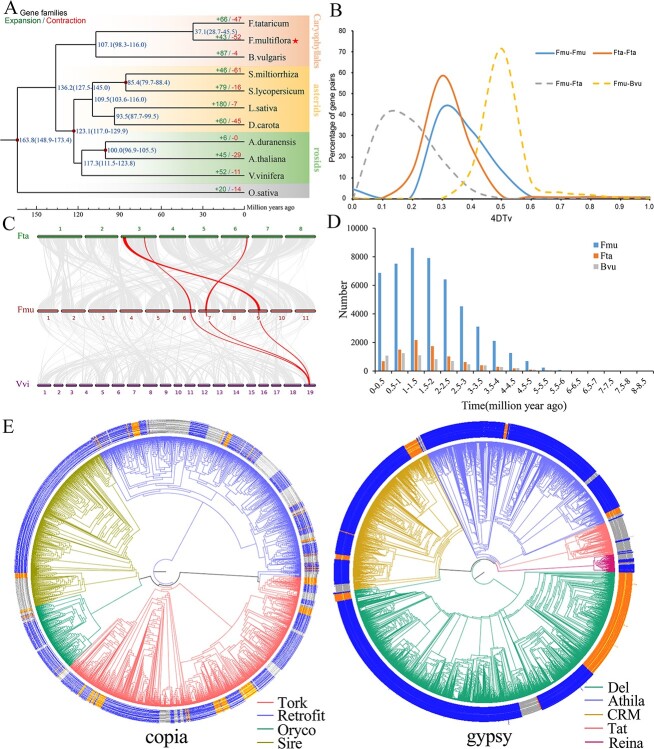
The evolution of the *Fallopia multiflora* genome. **A** Phylogenetic tree compiled from 955 single-copy genes from 11 plant species. Green indicates expansions of the gene family, whereas red indicates contractions of the gene family. Maximum Likelihood times (PAML) were used to estimate divergence. **B** The distribution of 4DTv is illustrated by colored lines. **C** Synteny analysis among the *F. multiflora* (Fmu)*, Fagopyrum tataricum* (Fta) and *Vitis vinifera* (Vvi) genomes. Grey links represent one-to-one syntenic homologues. **D** Distribution of insertion times for LTRs. **E** LTRs phylogenetic study in *F. multiflora* (blue), *F. tataricum* (orange) and *Beta vulgaris* (grey) genome. The trees were constructed by the neighbor-joining method using the Ty3/gypsy and Ty1/copia sequences.

The phenotypic diversification of plants is closely associated with gene family expansion and contraction [15, 16]. Thus, gene families showing expansion or contraction in 11 plant species compared with their ancestors were investigated. In the *F. multiflora* genome, 43 and 52 gene families were dramatically contracted and expanded, accordingly (Fig. 2A). Among the significantly expanded *F. multiflora* families, functional analysis revealed enrichment for several GO terms and KEGG pathways, including oxidoreductase activity, monooxygenase activity, metabolic pathways, biosynthesis of secondary metabolites, and those involved in specialized metabolism (e.g. stilbenoid, diarylheptanoid, and gingerol biosynthesis), likely reflecting the importance of these genes for the biosynthesis of phenolics and stilbenoids in *F. multiflora* (Figs S7 and S8, see online supplementary material).

Genes under positive selection usually contribute significantly to the adaptability of the species. To identify the genes possibly related to *F. multiflora* characteristics, we performed a positive-selection analysis on the *F. multiflora* genome. This identified 293 genes that had potentially undergone positive selection using the likelihood ratio test (*P* < 0.01, rate of false discovery <0.05) and then analysed their GO and KEGG enrichment. Positive-selection genes’ functions were primarily enriched in terms such as metabolic pathways, phenylalanine, tyrosine, and tryptophan biosynthesis, pantothenate and CoA biosynthesis, diterpenoid biosynthesis, secondary metabolite biosynthesis, and brassinosteroid biosynthesis (Fig. S9, see online supplementary material).

To investigate *F. multiflora* evolution, we found 955 single-copy orthologous gene families in 11 species and utilized them to design a phylogenetic tree. *F. multiflora* was found to be closely related to *F. tataricum* [in the same family (Polygonaceae)] using phylogenetic investigation and formed a clade with *B. vulgaris* (Fig. 2A), which supports Caryophyllales not belonging to the superasterids in the core eudicots, the Caryophyllales differentiation before the split of rosids and asterids [17, 18]. We determined that Caryophylalles were likely to have separated from other eudicots approximately 136 million years ago (Mya), while the common ancestor of *F. multiflora* and *F. tataricum* diverged from *B. vulgaris* ~ 107 Mya. Additionally, *F. multiflora* and *F. tataricum* diverged at ~37 Mya (Fig. 2A). These results indicated a close relationship between *F. multiflora* and *F. tataricum* and correspond with their previously assigned phylogenetic location into the same family according to morphological characteristics.

### WGD analysis

Whole-genome duplications play major roles in evolution due to their expansion of genomes [19]. To explore WGD events that took place within the evolution of *F. multiflora,* we started by analysing the four-fold degenerate synonymous sites of the third codon-position values (4DTv) (Fig. 2B) of duplicate gene pairs. We identified one peak in the 4DTv distribution at ~0.3 for all pairs of the syntenic genes in the *F. multiflora* genome, indicating the presence of a WGD in *F. multiflora* after it diverged from *B. vulgaris* but before divergence from *F. tataricum* (Fig. 2). This implies that *F. multiflora* and *F. tataricum* have a common ancestor who underwent a recent duplication event (~66 Mya). This WGD is independent of other WGDs occurring in asterid and rosid species [20] and is compatible with phylogenetic analysis results. Moreover, we found that *F. multiflora* did not undergo an additional WGD event following its divergence from *F. tataricum*.

Comparison of the *F. multiflora* genome with that of *F. tataricum* revealed that 91% of the *F. multiflora* gene models could be accommodated in syntenic blocks corresponding to a single *F. tataricum* region and covering 88% of the gene space of *F. tataricum*, among which 88% had one orthologous region in *F. multiflora* (Fig. S10, see online supplementary material). We then compared the *F. multiflora* genome with that of *V. vinifera*, demonstrating that 45% of the *F. multiflora* gene models were placed within syntenic blocks corresponding to a single region in *V. vinifera*. Additionally, *V. vinifera* gene models (12%) located within syntenic blocks possessed three orthologous regions in *F. multiflora*, 25% had two, and 27% had one (Fig. S11, see online supplementary material). The results of the intergenomic collinearity study were compatible with the WGD event for *F. multiflora*, as shown by a 1:1 syntenic association between *F. multiflora* and *F. tataricum* and a 1:3 syntenic relation between *F. multiflora* and *V. vinifera* (Fig. 2B and C).

Sequence aligment of homologs and searches of the Pfam database identified gene families potentially associated with stilbenoid biosynthesis in the six species (Table S11, see online supplementary material). The RS family copy numbers in the *F. multiflora* genome were consistent with those of grape, but higher than those of tartary buckwheat in the same family.

### Long terminal repeat (LTR) retrotransposon expansions result in large genome size

Repetitive sequences usually take possession of a substantial portion of plant genomes and are closely associated with variation in functional adaptation and genome size. We determined that 976.30 Mb of repetitive sequences occupied 67.69% of the assembled *F. multiflora* genome (Table S12, see online supplementary material). LTR retrotransposons accounted for the largest percentage of these repeated sequences and constituted 57.11% of the genome (Table S13, see online supplementary material).

The *F. multiflora* genome size (1.44 Gb) is much larger than those of the two closely related species [*F. tataricum* (489 Mb) and *B. vulgaris* (566.6 Mb)]. Although LTR transposable elements account for the majority of repetitive sequences in the *F. multiflora* genomes (Table S13, see online supplementary material), the relationship between LTRs and genome expansion remains unknown. *F. multiflora* has the maximum LTRs of ~461.6 Mb in comparison to the other two closely associated Caryophyllales species (*F. tataricum* of ~104.8 Mb and *B. vulgaris* of ~100.3 Mb). We found intact-LTRs and examined the insertion timings of all intact-LTRs in *F. multiflora* to determine the expanded LTRs’ history. Insertion-time analysis showed that LTR insertion is an unbroken process (Fig. 2D). We identified a total of 37 488, 7930, and 5254 intact-LTRs in *F. multiflora*, *F. tataricum*, and *B. vulgaris*, respectively (Table S14, see online supplementary material). Although we observed no significant LTR proliferation in the *B. vulgaris* genome, those of *F. multiflora* and *F. tataricum* showed significant LTR expansion in the near past (~0.5–2.5 Mya); however, the number of LTR insertions in *F. multiflora* was significantly greater than seen in *F. tataricum*, and the expansion period was longer (Fig. 2D). These results demonstrated that the LTR expansion happened recently in *F. multiflora* and may have contributed to the increased genome size in comparison with related species.

To further investigate the evolution of LTRs in *F. multiflora*, we analysed the phylogenies of LTR retrotransposons in three Caryophyllales plants. The majority of LTRs in the *F. multiflora* genome were Gypsy elements (19.6% of the genome), while just 4.6% contained Copia repeats. This was consistent with our observations in the *F. tataricum* and *B. vulgaris* genomes, where Gypsy elements were more abundant than Copia elements. The systematic evolutionary association of the Ty3/gypsy and Ty1/copia LTR superfamilies within these three species were then analysed. Ty1/copia elements were categorized into four broad evolutionary clades based on their presence in these species: Oryco, Retrofit, Sire, and Tork (Table S15, see online supplementary material). Then, all Ty3/gypsy elements were classified into five key evolutionary lineages within these three species: Athila, Tat, CRM, Del, and Reina (Table S16, see online supplementary material). We evaluated copy numbers and constructed Ty3/gypsy and Ty1/copia phylogenetic trees to have a better understanding of their expansion (Fig. 2E). The results indicated that Ty1/copia was not significantly amplified in *B. vulgaris*. Tat Ty3/gypsy expanded significantly, accounting for 44.46% of the total (Fig. 2E; Table S16, see online supplementary material). However, the Tat showed no expansion in *F. multiflora* and *F. tataricum*. In *F. tataricum*, the Del Ty3/gypsy demonstrated significant expansion, which constituted 77.29% of the total (Fig. 2E;Table S16, see online supplementary material), which was in keeping with the higher number of LTR insertions in *F. tataricum* over the previous 2 Mya. Additionally, Sire and Tork reflected the Ty1/copia superfamily’s significantly expanded lineage in *F. multiflora* and accounting for 40.71% and 33.62% (Fig. 2E; Table S15, see online supplementary material), and the Del and Athila of Ty3/gypsy both expanded significantly, accounting for 51.14% and 30.38%, respectively (Fig. 2E, Table S15, see online supplementary material). The four greatly expanded lineages may be responsible for the *F. multiflora* genome expansion (Figs. 2D and E; Tables S14–S16, see online supplementary material).

### Transcriptome and metabolome analyses

Next, we conducted a detailed transcriptome sequencing using different *F. multiflora* organs based on the high-quality genome, including roots, stems, and leaves (*n* = 3). Gene-expression analysis identified 4519 (including 1607 up-regulated and 2922 down-regulated genes), 986 (including 274 up-regulated and 712 down-regulated genes), and 1257 (including 376 up-regulated and 881 down-regulated genes) differentially-expressed genes (DEGs) in the pairwise comparisons of root versus leaves, root versus stems, and stems versus leaves, respectively (Fig. S12, see online supplementary material). Figs S13 and S14 (see online supplementary material) show the KEGG and GO annotations for these DEGs, whose functions were primarily enriched in metetabolic pathways and secondary metabolite biosynthetic pathways (especially stilbenoid, diarylheptanoid and gingerol biosynthesis, phenylalanine metabolism, phenylpropanoid biosynthesis, and flavonoid biosynthesis). A total of 228 DEGs were common to all three comparison groups (Fig. S15, see online supplementary material).

Additionally, we performed metabolome analysis on the roots, stems, and leaves (*n* = 6) of *F. multiflora* to correlate stilbenes metabolism with gene expression. A total of 1968 substances were identified of different organs from *F. multiflora* by untargeted ultra-performance LC–MS/MS metabolite profiling, of which 1323 compounds were detected in positive ion mode and 645 in negative ion mode. Moreover, the principal component analysis (PCA) revealed a clear separation between the aboveground samples (stems and leaves) and underground samples (roots), suggesting clearly distinct metabolite profiles in the aboveground and underground organs of *F. multiflora* (Fig. S16, see online supplementary material). We then determined the differentially expressed metabolites (DEMs) between pairs of samples (stems vs. roots, leaves vs. roots, and stems vs. leaves), which showed high numbers of DEMs between the compared samples (625, 693, and 325 DEMs, respectively) (Fig. S17, Tables S17–S22, see online supplementary material). These DEMs were of various classes, although stilbene-related molecules were enriched in these core-conserved metabolites (Table S23, see online supplementary material), including trans-THSG, polydatin, oxyresveratrol, resveratrol, and piceatannol. KEGG pathway enrichment of the DEMs was then performed to determine differences in their metabolic pathways (Fig. S18, see online supplementary material). Among the DEMs detected in all comparison groups, the most significant enrichments were seen in ‘Biosynthesis of secondary metabolites’; ‘Biosynthesis of stilbenes, diarylheptanes, and gingerols’; and ‘Phenylalanine metabolism’.

**Figure 3:**
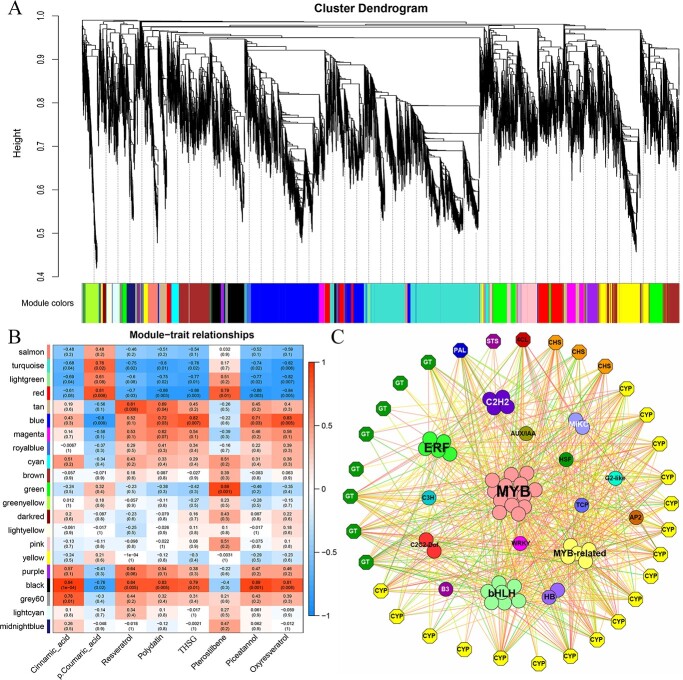
The outcomes of the network analysis. **A** Dendrogram illustrating modules found using the WGCNA and dendrogram showing clustering of expressed genes. **B** Module–trait relationships. **C** Association of transcription factors and genes. Genes associated with specific pathways are indicated using hexagons while transcription factors are shown as circles. Line color represents the type of correlation, with red indicating positive and blue indicating negative correlations. Darker colors indicate higher correlations.

In total, 17 unique modules with comparable gene expression patterns were evaluated using weighted gene co-expression network analysis (WGCNA), and similar genes were clustered into modules of the same color using the dynamic hierarchical tree cut approach (Fig. S19, see online supplementary material). Among the 17 modules, six (brown, green, turquoise, red, yellow, and Lightsteelblue1) contained >1000 genes, with the eigengenes of the root, stem, and leaf samples higher in the magenta, brown, and blue modules, respectively. Pearson correlations were then utilized to examine the association between module eigengenes and the abundances of stilbenes (Fig. 3), which comprised resveratrol, polydatin, trans-THSG, piceatannol, and oxyresveratrol. Correlation analysis revealed the biological significance of three modules: black, red, and green. Cinnamic acid (0.94), resveratrol (0.84), polydatin (0.83), trans-THSG (0.79), piceatannol (0.89), and oxyresveratrol (0.81) were closely related to the black module; the red module closely related to the content of *P*-coumaric acid (0.81), and the green module highly correlated with pterostilbene (0.89). These findings indicated that the genes in the three modules were strongly associated with stilbene accumulation in *F. multiflora*.

### Analysis of key enzyme genes and transcription factors associated with THSG biosynthesis

THSG is one of the earliest stilbenes found in *F. multiflora* and represents a derivative of resveratrol produced through hydroxylation and glycosylation [21]. Stilbenes are secondary metabolites of phenylpropanoids, which are composed of 1,2-diphenylethylene backbone. Several studies have suggested the derivation of stilbene biosynthesis from that of phenylalanine synthesis, and it is considered to be a competitive extension of the flavonoid branch [14, 22–24]. The most important step in stilbene synthesis involves STS-mediated catalysis of *P*-coumaroyl-CoA (one molecule) or cinnamoyl-CoA (one molecule) and malonyl-CoA (three molecules) [25–27]. Based on previously published protein sequences of key enzyme genes involved in stilbene biosynthesis from other plants, such as *A. thaliana*, *V. vinifera*, and *Polygonum cuspidatum,* we found 13 *PALs*, 13 *C4Hs*, 33 *4CLs,* and nine *STSs* in the *F. multiflora* genome (Table S24, see online supplementary material).

A previous study reported that UDP glycosyltransferases (UGTs) are involved in plant stilbene biosynthesis. The presence of forms of glycosylated resveratrol in *F. multiflora* suggests the initial synthesis of free resveratrol before its glycosylation by endogenous glycosyltransferases; therefore, *UGTs* may be related to the biosynthetic process of the glycoside products of stilbenes in *F. multiflora* [28–31]*.* In the *F. multiflora* genome, 321 putative genes encoding UGTs were identified. The expression patterns of UGT-associated genes were investigated based on WGCNA results (three modules, including black, red, and green). This indicated positive associations, either high or moderate, between UGT gene expression and the stilbene content in *F. multiflora* (Table S25, see online supplementary material)*.* In order to identify the functions of these eight potential UGTs, we further selected the UGTs with clear functions in Arabidopsis for phylogenetic analysis. The results show that four UGTs were identified as UGT72 (GT_evm.model.scaffold_287.161), UGT73 (GT_evm.model.scaffold_50.32), UGT80 (GT_evm.model.scaffold_32.33), and UGT89 (GT_evm.model.scaffold_676.68) families, respectively, and the other four cannot be classified into any of the known UGT families (Fig. S20, see online supplementary material). It is noteworthy that GT_evm.model.scaffold_287.161 belongs to the same family as the previously reported UGT involved in stilbene metabolism [32, 33].

Cytochrome P450s (CYP450s) are involved in some complex and diverse types of biosynthetic reactions, including continuous oxidation, hydroxylation reactions, and methyl or amino transfer of amines and their derivatives. There are many CYP450s associated with stilbene biosynthetic pathways in *F. multiflora* [34]. A phylogenetic study of 364 *CYP450s* revealed that the *CYP71*, *CYP72*, *CYP76*, *CYP82*, *CYP86*, *CYP94*, and *CYP96* superfamilies were enriched for more genes, with most of these genes taking part in the oxidative stress response [35] and the biosynthesis of terpenes [36, 37], indole alkaloids [38, 39], and sterols [40]. To identify potential cross-species *CYP450s*, we annotated a total of 12 *CYP450* genes involved in stilbene biosynthesis in *F. multiflora* based on WGCNA analysis (Table S25, see online supplementary material)*.* The 11 *CYP450* genes were most strongly expressed in roots, which was consistent with the accumulation pattern of stilbene glycosides in different *F. multiflora* organs. Notably, the 11 *CYP450* genes mainly belonged to the *CYP76*, *CYP82*, *CYP86*, and *CYP81* superfamilies based on phylogenetic analysis (Fig. S21, see online supplementary material). In some plants, the *CYP76*, *CYP82*, and *CYP81* superfamilies demonstrate hydroxylation functions [41–43]. These results suggested that 11 *CYP450* genes might play critical roles in stilbene biosynthesis in *F. multiflora*; however, the specific roles of these enzymes require further investigation.

The functions of *F. multiflora* transcription factors were then analysed. The genome contained 2539 transcription factors belonging to 89 different families (Table S26, see online supplementary material); the associations between these and genes associated with stilbene biosynthesis were investigated by correlations and the compilation of a gene regulatory network (Fig. 3C). This showed strong correlations between pathway genes and MYB, ERF, bHLH, WRKY, C2H2, and MYB-related transcription factors, with the MYB family including 14 of these. MYB transcription factors play key roles in plant metabolism, development, and stress responses, to both biotic and abiotic stress [44]. In *A. thaliana*, R2R3 MYB is divided into 25 subgroups (SG) according to phylogeny [44]. SG1–SG7 MYBs are involved in the synthesis of specialized metabolites with SG1 participating in cuticular wax synthesis of cuticular wax and stomatal control, while SG2s involved in phytoalexin (stilbene) synthesis, SG3s in lignification, and SG4s, SG5s, SG6s, and SG7s in phenylpropanoid, proanthocyanidin, anthocyanin, and flavonol biosynthesis, respectively [45]. To identify the subgroups of *F. multiflora* MYBs, a phylogenetic tree of the *A. thaliana* and *V. vinifera* MYB sequences was constructed. The phylogenetic tree results showed that evm.model.scaffold_628.78 and evm.model.scaffold_711.118 clustered with the sequence encoding SG3 MYB. In addition, five genes (evm.model.scaffold_38.1, evm.model.scaffold_383.144, evm.model.scaffold_501.154, evm.model.scaffold_707.47, evm.model.scaffold_53.36) clustered with the sequence encoding SG5 MYB (Fig. S22, see online supplementary material). It is noteworthy that MYB30 and MYB15 of *V. vinifera* can regulate the expression of STS, thus promotion of resveratrol synthesis [46, 47]. There are three MYB genes (evm.model.scaffold_70.96, evm.model.scaffold_180.45, evm.model.scaffold_168.46) of *F. multiflora* and MYB14 (VIT_07s0005g03340) converge into one branch (SG2), and three MYB genes (evm.model.scaffold_66.27, evm.model.scaffold_38.139, evm.model.scaffold_446.85) and MYB30 converge into one branch (SG1) (Figure S10, see online supplementary material). These results provide a reference for further investigation into MYB regulation of resveratrol synthesis in *F. multiflora*.

### Functional identification of *RS* genes in *F. multiflora*

STS is crucial for stilbene biosynthesis and is used as a substrate-precursor molecule that is present throughout the plant kingdom. A closely related enzyme, chalcone synthase (CHS), uses the same substrates and intermediates as STS but performs a divergent cyclization reaction to produce chalcone. STS and CHS belong to the PKS family [14], and because they share the same catalytic domain, the difference in their functions is mainly due to the difference in amino acids present in their respective active site or nearby regions. Based on sequence homology alignments, many plant STS sequences are likely to be incorrectly annotated as CHSs in various online databases [48–50].

Resveratrol is widely found in plants and currently represents the most intensively studied stilbene; however, few studies have investigated resveratrol modifications in the synthesis of stilbene derivatives [51, 52]. In transgenic *Arabidopsis*, overexpression of one *RS* gene from *P. cuspidatum* increases the accumulation of trans-piceid in the stilbene biosynthetic pathway and enhances resistance against *Colletotrichum higginsianum* [53]. RS is an STS, and resveratrol-forming *STS* genes have been identified in a variety of plant species, including mulberry [54], groundnut [55], and grapevine [50]. Unfortunately, there are few relevant reports related to the functions of *RS* and *CHS* in *F. multiflora* [56]. To investigate the stilbene biosynthetic capacity of *F. multiflora*, we mined its genome for *RS* and *CHS*.

**Figure 4:**
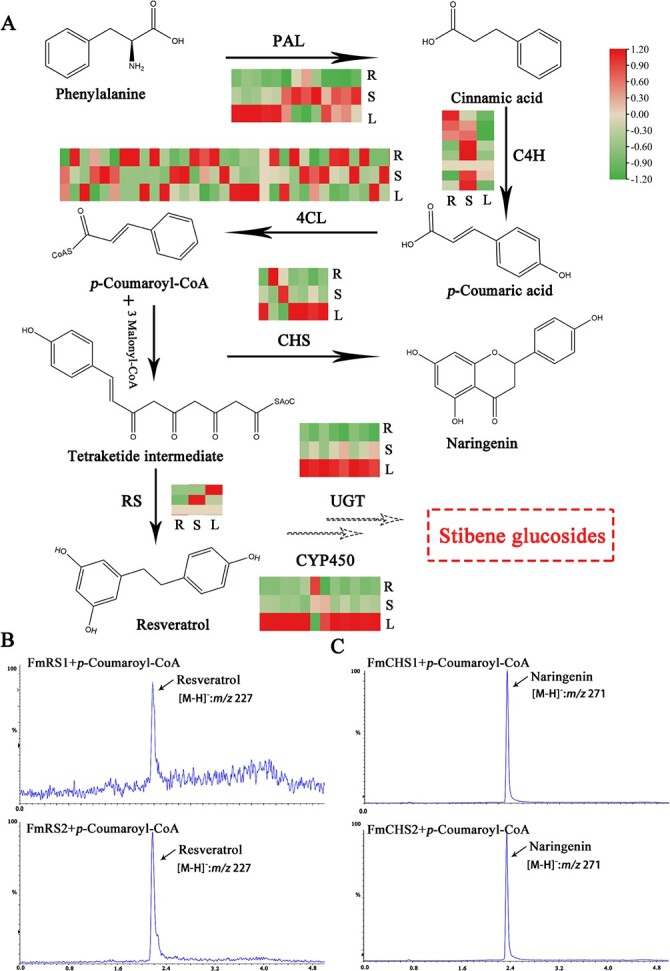
An overview of stilbene biosynthesis in *Fallopia multiflora* and functional verification of two RS genes and two CHS genes. **B** Representative UPLC-MS/MS chromatograms (MRM) of *FmRS1* and *FmRS2*. **C** Representative UPLC-MS/MS chromatograms (MRM) of *FmCHS1* and *FmCHS2*.

We ultimately identified the full-length sequences of three *RS* genes (Table S24, see online supplementary material) and named them *FmRS1*, *FmRS2*, and *FmRS3*. We did not detect the expression of *FmRS3* in any organ. Combined collinearity analysis and phylogenetic analysis identified *FmRS1* and *FmRS2* as tandem repeats on Chr2 (Fig. S23, see online supplementary material), with *FmRS1* closely related to *FmRS2* with 86.8% identity. To explore the activities of *FmRS1* and *FmRS2*, we cloned the coding sequences into pET28a for recombinant expression of *Escherichia coli*, followed by assays of activity using crude enzyme preparations (Fig. S24, see online supplementary material). As shown by LC–MS, *FmRS1* and *FmRS2* could convert one molecule of *p*-coumaroyl-CoA and three molecules ofmalonyl-CoA, to the same products (Fig. 4B; Figs S24 and S25, see online supplementary material) which were identified as resveratrol ([M-H]^−^: m/z 227 and 185). These findings identified *FmRS1* and *FmRS2* as new genes that exhibit STS activity in *F. multiflora*.

Additionally, we cloned seven full-length *CHS* genes identified in the *F. multiflora* genome, with subsequent phylogenetic analysis showing that *FmCHS1* and *FmCHS2* are closely related to *CHSs* from *P. cuspidatum* and highly expressed in different organs (Fig. S20 and Table S24; see online supplementary material). The other five *FmCHSs* (*FmCHS3–7*) clustered in the same branch and then clustered with the other *CHS* genes. We then cloned the seven *FmCHS*s into pET32a and used them to transform *E. coli* BL21 (DE3), ultimately finding that recombinant *FmCHS1* and *FmCHS2* exhibited PKS activity involving *p*-coumaroyl-CoA as starter CoA substrate in the presence of malonyl-CoA, resulting in naringenin chalcone ([M-H]^−^: m/z 271 and 151) (Fig. 4C; Figs S24 and S26, see online supplementary material). Notably, we detected no activity for the remaining five recombinant *FmCHSs* at any ratio of *p*-coumaroyl-CoA to malonyl-CoA.

## Discussion

The assembly and analysis of a high-quality reference-grade genome of *F. multiflora* provides valuable biological genetic information for the in-depth investigation of stilbenoid biosynthesis. This represents the first genome sequence of *Fallopia*. The *F. multiflora* genome is approximately 1.44 Gb in size anchored into 11 chromosomes, with 35 926 protein-coding genes. We identified a WGD in the *F. multiflora* lineage that happened following the lineage diverging from the sugar beet and before the lineage diverged from Tartary buckwheat. However, *F. multiflora* did not undergo lineage-specific WGD. This relatively larger genome may be related to the repetitive sequences and transposon elements compared with other phylogenetically related species such as Tartary buckwheat and sugar beet.

Polygoni Multiflori Radix and Polygoni Multiflori Caulis are important TCMs derived from the dried root tubers and stems of *F. multiflora*, respectively*.* However, the clinical effects of TCMs differ: Polygoni Multiflori Radix has the effect of moistening intestine detoxification, whereas Polygoni Multiflori Caulis mainly nourishes the mind, calms the spirit, dispels wind, and activates meridians [3]. Comparative metabolome analysis of *F. multiflora* from different organs revealed that the total metabolites of the underground parts (roots) differed significantly from the aerial parts (stems and leaves). We defined sets of DEGs for each pairwise comparison, which yielded 986 DEGs between roots and stems and 4529 DEGs between roots and leaves, all of which mainly participated in the biosynthesis of secondary metabolites, stilbenoids, diarylheptanoids, and gingerol biosynthesis. The synergistic influences of these genes and metabolites lead to variations in the clinical efficacy of the various medicinal parts of *F. multiflora*, and additional research of the pharmacology of relevant DEMs will more meticulously divulge the efficacies of different medicinal parts from the same source and provide novel insights into the investigation of these medicinal plants, including *Morus alba* L. [57, 58], *Lonicera japonica* Thunb. [59, 60], and *Albizia julibrissin* Durazz [61].

We then evaluated all candidate genes involved in stilbene synthesis in *F. multiflora*, and found that the essential genes responsible for stilbene biosynthesis were multiple-copy genes. We identified 13 *PALs*, 13 *C4Hs*, 33 *4CLs,* and three *RSs* and obtained 321 putative genes encoding *UGTs* and 364 *CYP450s.* To further investigate the functions of *UGT* and *CYP450* encoding enzymes that catalyze stilbene derivatization, based on the WGCNA data, we conducted a mining analysis of UGT and major CYP450 genes involved in the stilbene production pathway. We screened three modules of highly correlated genes, including eight *UGTs* and 11 *CYP450*s, all of which likely participate in stilbene biosynthesis in *F. multiflora* and are widely distributed across different chromosomes. Notably, we discovered *C4Hs*, *4CL*, *RSs*, *UGTs,* and *CYP450s* situated in close proximity to one another on chromosome 02 in the *F. multiflora* genome and reportedly involved in stilbene biosynthesis.

The stilbene resveratrol has been intenstively investigated [62] and has recently attracted increased attention along with the identification of key genes, such as *PAL*, *C4H*, *4CL*, and *RS*, encoding enzymes involved in its biosynthesis. *RS* genes encode enzymes (STSs) critical to resveratrol synthesis. A previous study identified 43 *RS* genes in grape, most of which have biological activity [63]. In the current investigation, we identified three *FmRS* genes in the *F. multiflora* genome, although they showed low expression in the roots, where resveratrol accumulates and similar to a pattern observed in *P. cuspidatum* [64]. Another study indicated that associations between stilbene contents and the expression of RS genes are not well understood [65]. Because posttranscriptional and posttranslational regulation occurs, analysis of transcription may not accurately reflect the ultimate level of protein expression. Moreover, resveratrol accumulation is regulated by not only related biosynthetic genes but also affected by transcription factors [66] and promoters [67, 68]. A previous report indicated that the catalytic efficiency (kcat/Km) of RS in *P. cuspidatum* is 2.4-fold higher than that in grape, which might explain the difference in resveratrol content among different species. However, the catalytic efficiency of RS in *F. multiflora* requires further investigation [69].

Interestingly, among the three *FmRS* genes identified in *F. multiflora*, two were tandem repeats (*FmRS1* and *FmRS2*), indicating that these might be critical for stilbene synthesis in *F. multiflora.* Assays of the recombinant enzymes showed that *FmRS1* and *FmRS2* have the function of catalyzing the production of resveratrol from malonyl-CoA and *p*-coumaroyl-CoA. Because CHS and RS have the characteristics of high homology, we performed functional validation experiments on CHS genes in *F. multiflora.* As expected, *FmCHS1* and *FmCHS2* have the function of catalyzing the *p*-coumaroyl-CoA and malonyl-CoA into naringenin chalcone.

In conclusion, we assembled a high-quality chromosomal-level genome for a member of the family Polygonaceae and identified diverse candidate genes responsible for stilbenoid biosynthesis in *F. multiflora*. These genes and genomic resources provide a foundation for further investigations into the biosynthesis, regulation, and transport of related metabolites.

## Materials and methods

### Plant materials

Perennial wild *F. multiflora* was originally collected from the Dashu Mountains, Hefei, Anhui Province, China. The whole plant was transplanted and stored for 4 years in the medicinal botanical garden of Anhui University of Chinese Medicine. Young leaves were placed in liquid nitrogen for DNA extraction. Seed, fruit, flower, leaf, stem, and root samples were collected, placed directly in liquid nitrogen for transcriptome sequencing and metabolites analysis. Prior to use, each sample was stored at −80°C.

### Evaluation of genome size

The characteristics of the genome were evaluated using K-mer frequency assessment [70]. Using the k-mer (k = 17) statistics, we calculated the genome size of *F. multiflora*, which used the modified Lander–Waterman algorithm. By the sequencing depth, the length of the reads was divided, and the peak value of the curve represented the overall sequencing depth. The following formula was then used to estimate the genome’s size: (N×(L–K + 1)–B)/D = G, where G denotes the size of the genome, D denotes the overall depth, B denotes the total number of K-mers with a lower frequency, K denotes the length of the K-mer (17 bp) [71], L represents the average read length and N demonstrates the total number of sequences reads. In addition, the genome size was estimated using flow cytometry. The nuclear DNA content was measured using a Partec CA II (Partec, Munster, Germany).

### Library construction and genome sequencing

DNA was extracted from young *F. multiflora* leaves using a DNAsecure Plant Kit (TIANGEN, Beijing, China) and a library construction kit was used to construct sequencing libraries with 350-bp insert sizes. After that, these libraries were sequenced on the Illumina HiSeq X platform (Illumina, San Diego, CA, USA). We used sheared DNA of at least 10 μg to construct SMRTbell libraries (20 kb inserts), which were sequenced by the PacBio Sequel platform.

We used DNA with a length of 50-kb for the construction of 10× Genomics libraries. DNA from young leaves was used for constructing the Hi-C library. The leaf tissue was fixed with foramdelyde, followed by lysis and chromatin digestion by the *Hind*III endonuclease. By eliminating proteins with protease, the molecules of DNA were released from the crosslinks. Finally, the DNA that had been purified was sheared into fragments of 350 bp to construct libraries [72], which were then sequenced on the Illumina HiSeq X platform.

### RNA sequencing

We extracted RNA from the different tissues with an RNAprep Pure PlantKit (TIANGEN, Beijing, China). In compliance with the recommendations of the manufacturer, the cDNA library was constructed by a NEBNext UltraRNA Library Prep Kit for Illumina (NEB) and sequenced on the Illumina HiSeq Xplatform.

### Genome assembly and quality assessment

PacBio SMRT long reads were used for *de novo* assembly with FALCON (https://github.com/PacificBiosciences/FALCON/) [73] and the initial assembly was polished using Quiver [74]. Then, errors in the Illumina sequences were corrected with Pilon [75]. After that, the Pruge_haplotig tool [76] was used to process genomic heterozygous regions to remove redundancy in the genomes using the default parameters. Then, 10× Genomics data were implemented to align to the assembly through BWA-MEM using default settings [77]. FragScaff was used to perform scaffolding [78]. Subsequently, we aligned the assembled scaffolds with the Hi-C sequencing data by BWA-MEM [77], and LACHESIS (http://shendurelab.github.io/LACHESIS/) were used to cluster the scaffolds onto chromosomes. BUSCO (http://busco.ezlab.org/) with the embryophyta odb10 database [79] and Core Eukaryotic Genes Mapping Approach (CEGMA) [80] were utilized to determine completeness. Further evaluation of the assembly was conducted using BWA-MEM mapping of the high-quality short paired reads [77].

### Repetitive sequence annotation

Transposable elements (TEs) in the *F. multiflora* genome were estimated using both *de novo*-based and homology-based methods. We employed Repeat Modeler (http://www.repeatmasker.org/RepeatModeler.html) [81], RepeatScout (http://www.repeatmasker.org/), and LTR_FINDER (http://tlife.fudan.edu.cn/ltr_finder/) [82] to build a *de novo* repeat library. Then we employed RepeatMasker (http://www.repeatmasker.org, Ver. 3.3.0) versus the Repbase TE library and RepeatProteinMask (http://www.repeatmasker.org/) versus the TE protein databank [83].

### Gene prediction and annotation

Protein-coding genes in the *F. multiflora* genome were identified employing de novo, transcriptome, and homolog prediction. Sequences of homologous proteins from five plant genomes (*A. thaliana, F. tataricum, Fagopyrum esculentum, Phytolacca americana*, and *V. vinifera*) were obtained from NCBI (https://www.ncbi.nlm.nih.gov/) and were aligned using tblastN with the *F. multiflora* genome assembly [84], with the parameters ‘E-value 1e-5’. The BLAST hits were conjoined using the Solar computer program [85]. Subsequently, we used GeneWise (https://www.ebi.ac.uk/Tools/psa/genewise) to determine the precise gene structure [86]. TopHat (http://ccb.jhu.edu/software/tophat/index.shtml, Ver. 2.0.8) and Cufflinks (http://cole-trapnell-lab.github.io/cufflinks/, Ver. 2.1.1) [87, 88] were performed to map RNA-seq information to the assembly. Pseudo-unigenes were created using Trinity by assembling the RNA-seq data and PASA (http://pasapipeline.github.io/) [89] was performed to estimate the gene models. For *ab initio* gene estimation methods, five *ab initio* gene estimation programs, including GENSCAN (http://genes.mit.edu/GENSCAN.html, Ver. 1.0), SNAP (http://korflab.ucdavis.edu/software.html), Augustus (http://augustus.gobics.de/, Ver. 2.5.5), geneid (http://genome.crg.es/software/geneid/), and GlimmerHMM (http://ccb.jhu.edu/software/glimmerhmm/, Ver. 3.0.1), were performed to estimate gene regions from the repeat-masked genome [90–93]. Finally, EVidenceModeler (EVM) (http://evidencemodeler.sourceforge.net/) was used for combining the gene model evidence from *ab initio* Cufflinks-set, PASA-T-set and Homo-set programs [94]. BLASTP (E-value: 1e-05) [95] were utilized to annotate the functions of protein-coding genes with SwissProt (http://web.expasy.org/docs/swiss-prot_guideline.html) and NR (ftp://ftp.ncbi.nih.gov/blast/db/). We used InterProScan (V4.8) and HMMER (http://www.hmmer.org/, Ver. 3.1) [96–99] to annotated protein domains through searching versus the InterPro (http://www.ebi.ac.uk/interpro/, Ver. 32.0) and Pfam (http://pfam.xfam.org/, Ver. 27.0) databases. Each gene of the GO (http://www.geneontology.org/page/go-database) terms was anticipated based on their InterPro or Pfam entries. KEGG database (http://www.kegg.jp/kegg/kegg1.html, release 53) was utilized to obtain the results of the pathway by BLAST (E-value 1e-05).

tRNAscan-SE software was used to identify the tRNA genes [100]. BlastN (E-value 1e-10) was performed to align to the rRNA sequences to predict the rRNA fragment. INFERNAL [101] predicted miRNA and snRNA genes with reference to the Rfam database (Ver. 9.1) [102].

### Phylogenetic analysis

Protein sequences of *A. thaliana, A. duranensis, V. vinifera, S. miltiorrhiza, S. lycopersicum, D. carota, L. sativa, B. vulgaris, F. tataricum*, and *O. sativa* were obtained from NCBI and aligned using BLASTP (E-value 1e-5). The sequences from 11 species were classified into paralogs and orthologs using OrthoMCL (http://orthomcl.org/orthomcl/) (inflation 1.5). After that, 955 single-copy genes were aligned by MUSCLE [103] and combined into a super-alignment matrix. A maximum-likelihood tree was constructed using RAxML (http://sco.h-its.org/exelixis/web/software/raxml/index.html) [104]. *O. sativa* was used as outgroups.

MCMCTree (http://abacus.gene.ucl.ac.uk/software/paml.html) (PAML) was used for prediction of divergence times [105]. The calibration time of divergence between *A.thaliana* and *A.duranensis* was 98.0–117.0Mya, *S. miltiorrhiza* and *S. lycopersicum* was 75.0–88.0 Million years ago (Mya), *A. thaliana* and *S. lycopersicum* was 111.0–131.0 Mya, *A. thaliana* and *O. sativa* was 148.0–173.0 Mya, according to the TimeTree database (http://www.timetree.org/). Finally, the CAFE program [106] was performed for determining the contraction and expansion of the gene families. Comparing *B. vulgaris* and *F. tataricum,* the single-copy genes from *F. multiflora* were identified using PAML branch-site models.

### Genome synteny and WGD

BLASTp (E-value 1e-5) was used to align the protein sequences in *F. multiflora* and *F. tataricum*. MCScanX (−a, −e:1e-5, −u:1, −s:5) was applied to ascertain syntenic blocks [107]. Finally, the 4DTv values of syntenic segments was calculated.

### Insertion time estimate of LTRs

We predicted LTR-RTs with LTRharvest [108] and LTRfinder [82]. The tRNAscan-SE [100] program predicted tRNA sequences with annotations using LTRdigest [109]. Then the sequences of the 5′ and 3′ LTRs werealigned by MUSCLE [103], and we calculated the nucleotide variations (λ) of the intact LTR-RTs. Then we calculated the DNA substitution rates (K) with the formula: K = –0.75ln(1–4λ/3). Finally, we estimated the insert time of LTR-RTs with the formula: T = K/2r (r = 1.3 × 10^−8^ per site and per year).

### Transcription factor analysis

Transcription factors were identified by ITAK (http://itak.feilab.net/cgi-bin/itak/index.cgi), and neighbor-joining trees were created in TreeBeST [110] using JTT and 1000 bootstraps. Co-expression networks of transcription factors and genes involved in stilbene synthesis were created using PCC in R, using Pearson correlation coefficients and cutoffs of of 0.7 and *P*-values <0.05 for significance. Cytoscape was used to display the network connections.

### Metabolome analysis

The lyophilized plant tissues were individually ground using a Mixer Mill MM 400 (Retsch GmbH, 42 781 Haan, Germany), and we extracted approximately 100 mg samples and resuspended them with 500 μL prechilled (80%) aqueous methanol and formic acid (0.1%) by the well vortex. Following centrifugation (15 000 *g* for 20 min), the supernatants were diluted with LC–MS grade water to a final concentration of 53% aqueous methanol. Then the solutions were centrifuged (15 000 *g* for 20 min), and we pooled and filtered the supernatants with a membrane (0.22 μm) and injected into the UPLC-MS/MS system. The UPLC-MS/MS analysis was carried out utilizing a Vanquish UHPLC system (ThermoFisher, Germany) coupled with an Orbitrap Q Exactive™ HF mass spectrometer (Thermo Fisher, Germany). Samples were loaded onto a Hypesil Goldcolumn with a diameter of 100 × 2.1 mm and a length of 1.9 μm utilizing a linear gradient of 17 min at a 0.2 mL/min flow rate. The eluents for the positive polarity mode were eluent A (FA in Water of 0.1%) and eluent B (Methanol), and eluent A (ammonium acetate of concentration 5 mM, pH 9.0) and eluent B (Methanol) were employed for the negative polarity mode. QExactive™ HF mass spectrometer was run in polarity mode (negative/positive) at 3.2 kV, 320°C capillary temperature, 40 arb flow rate of sheath gas, and 10 arb aux gas flow rate. We performed peak picking, peak alignment, and quantization for each metabolite using the Compound Discoverer 3.1 (CD3.1, ThermoFisher). In order to predict the molecular formula, we used normalized data derived from molecular ion peaks, as well as additive and fragment ions. The peaks were subsequently matched with the mzCloud (https://www.mzcloud.org/), MassList, and mzVault databanks to attain accurate qualitative and related quantitative achievements.

### Gene cloing and functional characterization of *FmCHS/RS* genes from *F. multiflora*

Total RNA was extracted from *F. multiflora* roots using TRIzol (TaKaRa, China). PrimeScript II 1st Strand cDNA Synthesis Kit (TaKaRa, China) was employed for constructing first-strand cDNA from the whole RNA. Primers were designed for two *FmCHS*s and two *FmRS*s based on sequences from the genome data (Table S27, see online supplementary material). *FmCHS1*, *FmCHS2*, *FmRS1,* and *FmRS2* sequences were individually cloned into the pET32a and pET28a vector and next converted into *E. coli* Trans1-T1. After that, the recombinant plasmids were put into *E. coli* BL21 (DE3) to express the proteins. Isopropyl β-D-thiogalactoside (IPTG) od concentration 0.8 mM was added when the cell culture’s OD_600_ value (grown at 37°C) reached 0.4 ~ 0.6. After 12 h of incubation with shaking at 20°C, the bacterial suspension was centrifugated to remove the supernatant, and then 1 mL of His Buffer A was added to resuspend. The proteins were obtained after ultrasonic in ice for 15 min, followed by 4°C centrifugation. The procedure of *in vitro* enzymatic assays were modified from Lu *et al.* [56]. Generally, starter Coenzyme A (150 μM), malonyl-CoA (280 μM), 0.1 M potassium phosphate with a pH of 7.6 and crude enzyme (100 μL) were included in a 500 μL catalytic reaction system. After incubation 1 h at 60°C, the mixtures were extracted three times with ethyl acetate (250 μL) and centrifuged at 12000 *g* for 10 min. The supernatant obtained was dehydrated with nitrogen gas and then dissolved in methanol (100 μL) for UPLC-MS/MS analysis.

A AB Sciex QTRAP 5500 system (AB Sciex, USA) with a ACQUITY UPLC® BEH C_18_ column of 1.7 μm, 2.1 × 100 mm was used for UPLC-MS/MS detection. The column was used at 40°C, mobile phases A and B were formic acid of 0.1% aqueous solution (A) and acetonitrile (B), respectively, at a 0.4 mL/min flow rate. The elution gradient was performed as follows: 5% (0 ~ 1 min) B; 5% ~ 50% (1 ~ 2 min) B; 50% ~ 75% (2 ~ 3 min) B; 90% ~ 5% (3 ~ 4 min) B; 5% (4 ~ 5 min) B. The MS was used in negative ion mode for Multiple Reaction Monitoring (MRM), with the following conditions: ion spray voltage (4.5 kV); gas temperature (500°C); ion source gas (45 psi); curtain gas (40 psi).

## Acknowledgments

This study was funded by the National Natural Science Foundation of China (No. 81973432), the Major Increase and Reduction Project at the Central Level (No. 2060302), Innovation Team and Talents Cultivation Program of National Administration of Traditional Chinese Medicine (ZYYCXTD-D-202005), and CAMS Innovation Fund for Medical Sciences (2019-I2M-5-065).

## Author contributions

Y.Z., H.P. and L.H. conceived and supervised the study. Y.Z., Q.F., and Y.Q. collected the samples. Z.Z. carried out comparative genomics analysis and phylogenetic analysis. Z.Y., L.Z., and Z.T. contributed to molecular experiments and bioinformatics analysis. Y.Z. and M.Y. wrote the paper. S.C. prepared the supplementary figures and tables. Y.Y., L.H., and H.P. revised the paper. All authors read, edited, and approved the final paper.

## Data availability

The data described in this work is available upon reasonable request to the corresponding author. The raw genome sequencing data, Hi-C data, RNA-seq data, and genome assembly of *F. multiflora* in this study have been submitted to the NCBI BioProject database under accession number PRJNA793351. Other data, such as the assembled genome sequence and gene structure annotation are available at the FigShare database (10.6084/m9.figshare.19720381).

## Conflict of interests

The authors declare that they have no known conflicts of interest.

## Supplementary data

Supplementary data is available at *Horticulture Research* online.

## Supplementary Material

Web_Material_uhad047
