# Supplementary material for: The first chromosome-level Fallopia multiflora genome assembly provides insights into stilbene biosynthesis
Source: Hortic Res. 2023 Mar 15;10(5):uhad047. doi: 10.1093/hr/uhad047 (PMC10194901; doi:10.1093/hr/uhad047)
Supplement: Web_Material_uhad047 [file web_material_uhad047.zip › revised Supporting_Information.docx]

**Supplementary Information**

The first chromosome-level *Fallopia multiflora* genome assembly provides insights into stilbene biosynthesis

**Supplemental Figures**

**Figure S1. Evaluation of the genome size of *Fallopia multiflora* by flow cytometry (A, B) and 17-mer analyses (C, D).** **A.** DNA content histogram of control leaf sample of *F. multiflora*. **B.**DNA content histogram of experimental leaf sample of *F. multiflora*. **C.** Frequency distribution of KMER = 17 depth and number of k-mer in *F. multiflora* genome. **D.** Frequency distribution of KMER = 17 depth and species number of k-mer in *F. multiflora* genome.

**Figure S2. Hi-C intra-chromosomal contact map for the genome assembly (2n=22) using LACHESIS**

**Figure S3. Distribution of genes in different species.**

**Figure S4. Common and unique gene families of *Fallopia multiflora*, *F. tataricum* and *B. vulgaris*.**

**Figure S5. GO enrichment analysis of specific gene family of *Fallopia multiflora*.**

**Figure S6. KEGG enrichment analysis of specific gene family of *Fallopia multiflora*.**

**Figure S7. GO enrichment of significantly expanded gene families in *Fallopia multiflora* genome.**

**Figure S8. KEGG enrichment of significantly expanded gene families in *Fallopia multiflora* genome.**

**Figure S9. KEGG enrichment of positive selection genes in *Fallopia multiflora* genome.**

**Figure S10. Summary of the syntenic analysis between Fmu and Fta. Biologically independent sample (n=1).**

**Figure S11. Summary of the syntenic analysis between Fmu and Vvi. Biologically independent sample (n=1).**

**Figure S12. Volcano maps of differentially expressed genes.** The red dot indicates differential expression genes that have been up-regulated., the green dot indicates genes whose expression is down-regulated and the blue indicates the non-differential expression genes.

**Figure S13. GO function classification of differentially expressed genes.**

**Figure S14. KEGG enrichment assessment of differentially expressed genes.**

**Figure S15. Venn diagram of differentially expressed genes.**

**Figure S16. Principal component analysis of metabolites in different organs.** (**A**: negative ion mode, **B**: positive ion mode)

**Figure S17. Volcano map of different metabolite levels.** (**A**: negative ion mode. **B**: positive ion mode)

**Figure S18. KEGG enrichment of different metabolite levels.**

**Figure S19. The outcomes of the network assessment of the gene.** (**A**) Dendrogram illustrating modules identified using WGCNA and dendrogram showing clustering of expressed genes; (**B**) The number of genes that are contained within each module; (**C**) Each sample's module eigengenes are displayed as a heat map.

**Figure 20. Phylogenetic tree of the UGT gene family in *Fallopia multiflora* (black) and *Arabidopsis thaliana* (grey).** The UGT genes screened from *F. multiflora* are labelled in red.

**Figure** **S21. Phylogenetic tree of the CYP gene family in *Fallopia multiflora* (blue) and *Arabidopsis thaliana* (grey).** The CYP genes screened from *F. multiflora* are labelled in red.

**Figure S22. Phylogenetic tree of the MYBs in *Fallopia multiflora* (black), *Vitis vinifera* (blue) and *Arabidopsis thaliana* (grey).** The MYB screened from *F. multiflora* are labelled in red.

**Figure S23. Phylogenetic tree of *FmCHSs* and *FmRSs* and other plant homologous proteins.**

**Figure S24. SDS-PAGE analysis of the *FmCHS1*, *FmCHS2*, *FmRS1*, and *FmRS2* proteins in *Escherichia coli* BL21(DE3).**

**A** M: Standards for the molecular weight of proteins;1: IPTG-induced *E. coli* BL21(DE3)/pET-32a cell lysate; 2: Total cell extract of *E. coli* BL21(DE3)/pET-32a-FmCHS1 without IPTG induction; 3: IPTG-induced *E. coli* BL21(DE3)/pET-32a-FmCHS1;4: Total cell extract of *E. coli* BL21(DE3)/pET-32a-FmCHS2 without IPTG induction; and 5: IPTG*-*induced *E. coli* BL21(DE3)/pET-32a-FmCHS2.

**B** M: Standards for the molecular weight of proteins;1: IPTG-induced *E. coli* BL21(DE3)/pET-28a cell lysate; 2: Total cell extract of *E. coli* BL21(DE3)/pET-28a-FmRS2 without IPTG induction;3: IPTG-induced *E. coli* BL21(DE3)/pET-28a-FmRS2;4: Total cell extract of *E. coli* BL21(DE3)/pET-28a-FmRS1 without IPTG induction; and 5: IPTG-induced *E. coli* BL21(DE3)/pET-28a-FmRS1.

**Figure S25. UPLC-MS/MS analysis of reaction products formed by *FmRS1* (C), and *FmRS2* (D). Substrates are p-coumaroyl-CoA and malonyl-CoA. A (pET28a plus buffer) and B (Resveratrol standard plus buffer) are control groups.**

**Figure S26. UPLC-MS/MS analysis of reaction products formed by *FmCHS1* (C), and *FmCHS2* (D). Substrates are p-coumaroyl-CoA and malonyl-CoA. A (pET32a plus buffer) and B (Naringenin standard plus buffer) are control groups.**

**Supplemental Tables**

**Table S1. Statistics of genome characteristics of *Fallopia multiflora* according to 17-mer analyses.**

**Table S2. Summary of sequenced paired-end libraries for the *Fallopia multiflora* genome**

**Table S3. Statistics of chromosome length following Hi-C assisted assembly**

**Table S4. Hanging rate of the *Fallopia multiflora* genome**

**Table S5. Statistics of genomic reads coverage of *Fallopia multiflora***

**Table S6. Estimation of *Fallopia multiflora* genome by CEGMA and BUSCO results**

**Table S7. Statistical results of the *Fallopia multiflora* genome's gene structure prediction**

**Table S8. Functional annotation of the *Fallopia multiflora* genome**

**Table S9. Non-coding RNAs of *Fallopia multiflora* genome**

**Table S10. Genes utilized in each species for gene family clustering**

**Table S11. Copy number variation of genes involved in the stilbenoid biosynthesis in the F. multiflora and five other plant species.**

**Table S12. Statistical outcomes of repetitive sequences of *Fallopia multiflora* genome**

**Table S13. Statistical outcomes of repetitive sequences classification of *Fallopia multiflora* genome**

**Table S14. Summary of intact LTRs with at least one protein-coding genes in *Fallopia multiflora***

**Table S15. The proportion (%) of lineages in Ty1/copia super family**

**Table S16. The proportion (%) of lineages in Ty3/gypsy super family**

**Table S17. Differential metabolites of Leaf vs Root under negative ion mode**

**Table S18. Differential metabolites of Leaf vs Root under positive ion mode**

**Table S19. Differential metabolites of Stem vs Leaf under negative ion mode**

**Table S20. Differential metabolites of Stem vs Leaf under positive ion mode**

**Table S21. Differential metabolites of Stem vs Root under negative ion mod**

**Table S22. Differential metabolites of Stem vs Root under positive ion mod**

**Table S23. Top 20 metabolites in different comparison groups**

**Table S24. RPKM expression of key enzyme genes in the stilbene biosynthesis pathway in different organs**

**Table S25. Mining and expression analysis of UGT and CYP450 genes in the stilbene biosynthesis pathway of *Fallopia multiflora***

**Table S26. Statistics of transcription factors in Fallopia multiflora genome.**

**Table S27. Forward and reverse primer sequences used in this study**
